# Genome-wide annotation of protein-coding genes in pig

**DOI:** 10.1186/s12915-022-01229-y

**Published:** 2022-01-25

**Authors:** Max Karlsson, Evelina Sjöstedt, Per Oksvold, Åsa Sivertsson, Jinrong Huang, María Bueno Álvez, Muhammad Arif, Xiangyu Li, Lin Lin, Jiaying Yu, Tao Ma, Fengping Xu, Peng Han, Hui Jiang, Adil Mardinoglu, Cheng Zhang, Kalle von Feilitzen, Xun Xu, Jian Wang, Huanming Yang, Lars Bolund, Wen Zhong, Linn Fagerberg, Cecilia Lindskog, Fredrik Pontén, Jan Mulder, Yonglun Luo, Mathias Uhlen

**Affiliations:** 1 Department of Protein Science, Science for Life Laboratory, KTH-Royal Institute of Technology, Stockholm, Sweden; 2 Department of Neuroscience, Karolinska Institutet, Stockholm, Sweden; 3 Department of Immunology, Genetics and Pathology, Uppsala University, Uppsala, Sweden; 4 BGI-Shenzhen, Shenzhen, China; 5 Lars Bolund Institute of Regenerative Medicine, Qingdao-Europe Advanced Institute for Life Sciences, BGI-Qingdao, Qingdao, China; 6 Department of Biomedicine, Aarhus University, Aarhus, Denmark; 7 Steno Diabetes Center Aarhus, Aarhus University Hospital, Aarhus, Denmark; 8 MGI, BGI-Shenzhen, Shenzhen, China

**Keywords:** Annotation, Protein-coding genes, Genome-wide, Transcriptome, Gene expression, Tissue expression profile

## Abstract

**Background:**

There is a need for functional genome-wide annotation of the protein-coding genes to get a deeper understanding of mammalian biology. Here, a new annotation strategy is introduced based on dimensionality reduction and density-based clustering of whole-body co-expression patterns. This strategy has been used to explore the gene expression landscape in pig, and we present a whole-body map of all protein-coding genes in all major pig tissues and organs.

**Results:**

An open-access pig expression map (www.rnaatlas.org) is presented based on the expression of 350 samples across 98 well-defined pig tissues divided into 44 tissue groups. A new UMAP-based classification scheme is introduced, in which all protein-coding genes are stratified into tissue expression clusters based on body-wide expression profiles. The distribution and tissue specificity of all 22,342 protein-coding pig genes are presented.

**Conclusions:**

Here, we present a new genome-wide annotation strategy based on dimensionality reduction and density-based clustering. A genome-wide resource of the transcriptome map across all major tissues and organs in pig is presented, and the data is available as an open-access resource (www.rnaatlas.org), including a comparison to the expression of human orthologs.

**Supplementary Information:**

The online version contains supplementary material available at 10.1186/s12915-022-01229-y.

## Background

An important part of the functional genome annotation is to explore the body-wide expression patterns of all protein-coding genes across all major cell types, tissues, and organs. This allows the classification of proteins based on the expression of the corresponding protein-coding genes. For the human genome, such annotation has led to the annotation first with regard to tissue specificity based on the relative transcriptome levels across all major tissues [1], and secondly, with regard to tissue distribution, showing the fraction of tissues with detectable expression corresponding to a given gene [2]. Both these genome-wide annotation tools are useful, but require arbitrary cut-offs; hence, the resulting classification depends on a decision regarding what is relevant fold changes and detection limits for the underlying transcriptomics data.

We have therefore explored various algorithms for categorizing genes utilizing dimensionality reduction and density-based clustering to classify genes based on similarity of expression patterns across all major tissues and organs. We present, for the first time, a new approach for annotation of genomes based on Uniform Manifold Approximation and Projection (UMAP) clusters followed by functional Gene Ontology (GO) analysis and a tissue specificity analysis, combined with manual curation. The new approach for genome annotation has been performed on the pig transcriptome based on a comprehensive analysis of all major tissues and organs in the Bama minipig, a strain broadly used in biomedical research [3–5].

Pigs have become an attractive large animal model system in pharmacology [6], toxicology [7], and diseases [8] and as a model system in pharmacological, immunological, and other biomedical applications [9–11]. In addition, pigs are interesting as potential donors for organ transplantation [8, 12]. However, in contrast to more widely used model organisms, genomic, transcriptomic, and proteomic characterizations of pigs are limited, although important updates have been made recently improving gene annotations and genome coverage [13–20]. We here present a new UMAP-based classification scheme, called Tissue Expression Clustering, for genome-wide annotation based on body-wide expression patterns. The results are presented as an open-access resource with transcriptome analysis of all protein-coding genes across all major pig tissues and organs (www.rnaatlas.org).

## Results

### Body-wide transcriptomics analysis of the pig

**Fig. 1:**
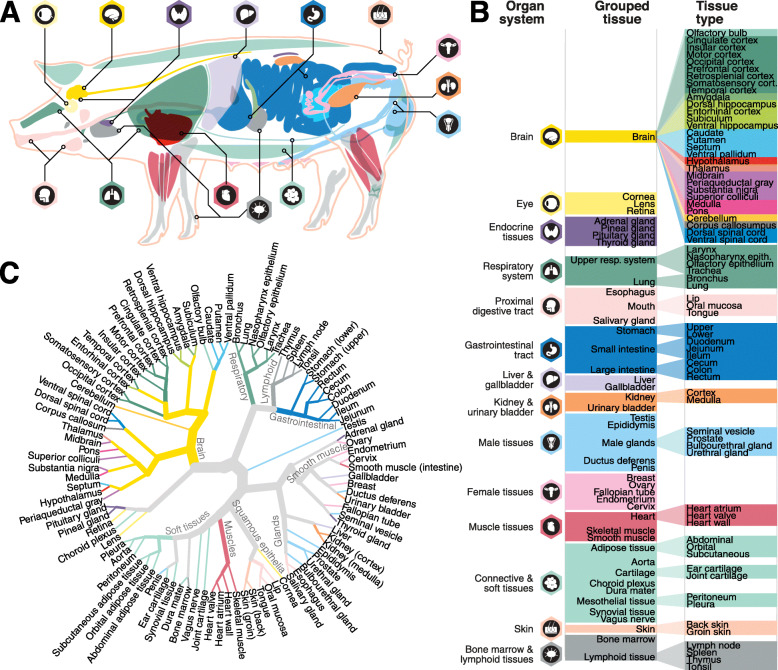
Whole-body expression analysis of the pig. **A** Organ schematic drawing of the pig body, following the established color code. **B** The 98 tissue types analyzed from the Bama minipig are grouped into 44 grouped tissues, each belonging to one of 14 organ systems. **C** Circular dendrogram based on Ward’s criterion on pairwise Spearman correlation between tissue types. Branch lengths have been scaled to reduce visual complexity. A selection of branches is annotated based on common biological features

To generate a body-wide expression atlas of the porcine protein-coding genes, 350 samples representing 98 different tissues and 14 organ systems (Fig. 1A) were collected from four young adult (two males and two females, 1-year-old) Bama minipigs. The 98 tissues were grouped into 44 main organ/tissue types based on shared developmental, functional, and/or anatomical properties (Fig. 1B). The 30 tissue types that represent the central nervous system were included in Sjöstedt et al. [21] comparing expression profiles across human, pig, and mouse brains. The protein-coding expression data of the pig brain is also integrated into the Human Protein Atlas (HPA) Brain section.

The dissection accuracy of the tissue samples was confirmed by histological analysis of adjacent tissue. Samples were sequenced with an average depth of 165.5 million reads (Additional file 2), and read counts were normalized (protein-coding transcript per million (pTPM) for visualization, and normalized expression (NX) for gene classification) for all 22,342 protein-coding genes. In total, 22,007 (98.5%) genes were detected (NX > 1) in at least one tissue type, ranging from 13,607 to 16,867 genes detected per tissue type (Additional file 1: Fig. S1A). Highly specialized tissue types, such as the lens and joint cartilage, express fewer genes, whereas tissues composed of many different cell types (e.g., testis and brain) express the highest number of genes, in line with results from human tissues [1, 22, 23].

To further investigate similarities in global transcriptome profiles between tissues, Spearman correlation was used in a pairwise correlation heatmap for the 44 grouped tissues (Additional file 1: Fig. S1B). The heatmap with a body-wide representation of all tissues and organs shows that testis and the various brain samples have the most divergent global expression profiles similar to the pattern in human tissues [1, 2]. The corresponding dendrogram based on the global transcriptomics profile across all protein-coding genes (Fig. 1C) demonstrates that related tissues cluster together, including tissues of the respiratory system, immune system, gastrointestinal tract, muscle tissues, and the nervous system. In general, closely clustering tissues often share germ layer origin, functions, and/or cellular composition, e.g., skin, mouth tissues, and cornea all include ectoderm-derived squamous epithelium [24, 25]. The esophagus, although containing squamous epithelium, revealed a high degree of similarity with the salivary gland and other secretory tissues due to the presence of esophageal glands [26]. Neuroectoderm-derived tissues such as brain tissues, pituitary gland, pineal gland, and retina cluster into one major branch (Fig. 1C and Additional file 1: Fig. S1B). The mesoderm-derived tissues, including all soft tissues, and skeletal and cardiac muscles are clustered closely. The endoderm-derived tissues including respiratory tissues (i.e., lung, bronchus, trachea, larynx) and gastrointestinal tissues are clustered together. In contrast, tissues composed of major cell types originating from different germ layers are clustered between the major germ layers, such as glands and reproductive tissues. The testis, with a large enrichment of germ cells, is clustered separately. Similar clustering patterns of tissues per germ layer has previously been described [27], including in pig specifically [15].

**Fig. 2:**
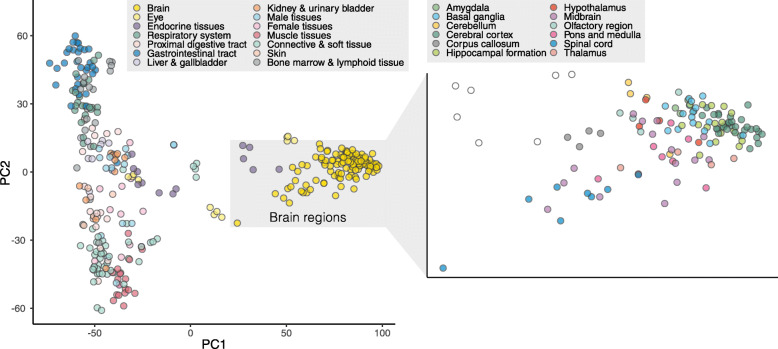
Principle component analysis (PCA) plot showing the relation and clustering of all tissue samples. Brain samples are shown in more detail in the zoom-in box (right)

The genome-wide expression profiles were investigated for all the 350 individual samples using dimensional reduction analysis and the results for principal component analysis (PCA) are shown in Fig. 2 and using UMAP in Additional file 1: Fig. S2A. The analysis shows that tissue types with related functions share similar global expression profile and that the brain samples have a unique expression pattern compared to peripheral tissues. The 30 brain subregions cluster according to the basic organization of the brain, with the spinal cord and brainstem together with corpus callosum and other white matter-rich regions, separated from neuronal rich cerebellum and cortical areas (Fig. 2). The shared developmental neuroectoderm origin between the brain, endocrine tissues, and retina [28, 29] is also seen in the global expression comparison. Additionally, tissues from the gastrointestinal (GI) tract show similarity to lymphoid tissues, possibly explained by local germinal centers in GI, and specialized GI immune cells [30]. The respiratory system is found close to the GI tract and lymphoid tissues, due to the presence of mucus-secreting goblet cells (also found in GI) and respiratory tract-associated immune cells [31]. A correlation analysis showed a high correlation between samples of the same tissue type, with an average Spearman correlation of 0.96, ranging from 0.89 to 0.99, depending on tissue type (Additional file 1: Fig. S2B).

### Genome-wide annotation of the protein-coding genes

To generate an overview of the body-wide distribution and specificity of pig genes, the gene classification approach used in the Human Protein Atlas program was adapted to classify all 22,342 porcine protein-coding genes as described in Additional file 3 [1, 2], and exemplified in Additional file 1: Fig. S3D. The categories of tissue enriched, group enriched, and tissue enhanced are collectively termed tissue elevated. The specificity categorization shows that 13,372 genes have elevated expression in one or more tissues, out of which 3085 genes show enriched expression (Additional file 1: Fig. S3A and S3C). Genes with elevated expression are as expected mostly found in tissues with highly specialized cells, such as the brain (*n* = 2930), testis (*n* = 2,718), and lymphoid tissues (*n* = 1,360) (Additional file 1: Fig. S3B). Whereas tissue types composed of large proportions of common structures and cell types have lower number of genes with elevated expression, such as smooth muscle-rich tissues or soft tissues (e.g., aorta and adipose tissues).

**Fig. 3:**
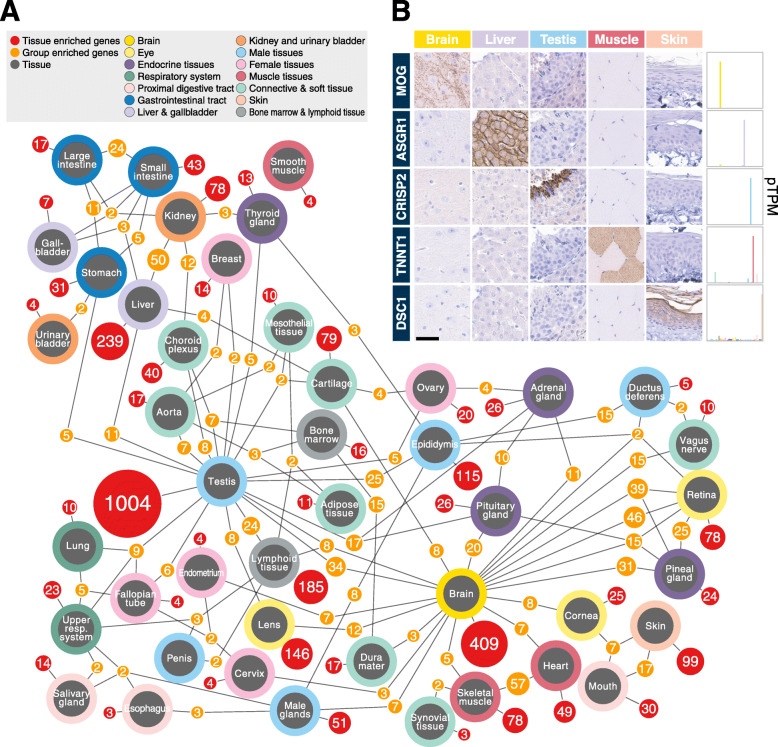
Gene classification based on tissue expression. **A** Network plot indicating the number of genes with tissue or group enriched expression for combinations of tissue types (tissues: gray nodes; tissue enriched: red nodes; group enriched: orange nodes). Nodes were filtered based on rules listed in “Methods” section to reduce visual complexity. **B** Immunohistochemical (IHC) staining (left) and RNA expression (right) of tissue enriched genes: MOG (Brain), ASGR1 (liver), CRISP2 (testis), TNNT1 (skeletal muscle), and DSC1 (skin)

A network plot (Fig. 3A) was constructed to visualize commonalities between tissues in terms of tissue and group enriched genes across all the tissues and organs analyzed here. Most tissue enriched genes are found in the testis (*n* = 1004) followed by the brain (*n* = 409) and liver (*n* = 239) similar to the corresponding analysis in the human body [2]. Most group enriched genes are found between the heart and skeletal muscle (*n* = 57) and between the kidney and liver (*n* = 50). The data has been published in a new open-access resource called the Pig RNA Atlas (www.rnaatlas.org), to allow researchers to explore the list of genes corresponding to the various tissues and organs. Furthermore, analysis of tissue distribution highlighted 1046 genes to be detected in a single tissue type (Additional file 1: Fig. S3A), out of which a large fraction was also classified as testis enriched. The highly specific expression of the testis is due to the testis-specific Sertoli and germ cells and has previously been described in human [2], pig [15, 32], macaque [33], and mouse [34]. In contrast, a large portion of the genes is classified as low tissue specificity and detected in all tissues (*n* = 7699), and this set of genes is also interesting to study further.

To confirm and further explore expression profiles observed in pig tissues at the protein level, we stained tissues with antibodies for visualization of proteins corresponding to genes classified as tissue enriched, in terms of location and distribution (Fig. 3B). The examples include the brain enriched Myelin oligodendrocyte glycoprotein (MOG), a protein detected in oligodendrocytes and myelin sheets in the brain; the liver enriched Asialoglycoprotein receptor 1 (ASGR1) which is a liver transmembrane protein detected in hepatocytes; the testis enriched Cysteine-rich secretory protein 2 (CRISP2) detected in spermatids; the skeletal muscle enriched Troponin T1 (TNNT1) detected in the slow muscle fibers; and skin enriched Desmocollin 1 (DSC1) a desmosomal cadherin detected in the membrane of keratinocytes. In all cases, the good agreement between the RNA expression and protein detection supports the approach to use RNA as proxy for mapping protein profiles in tissue.

### New genome-wide classification of expression profiles based on UMAP dimensionality reduction

**Fig. 4:**
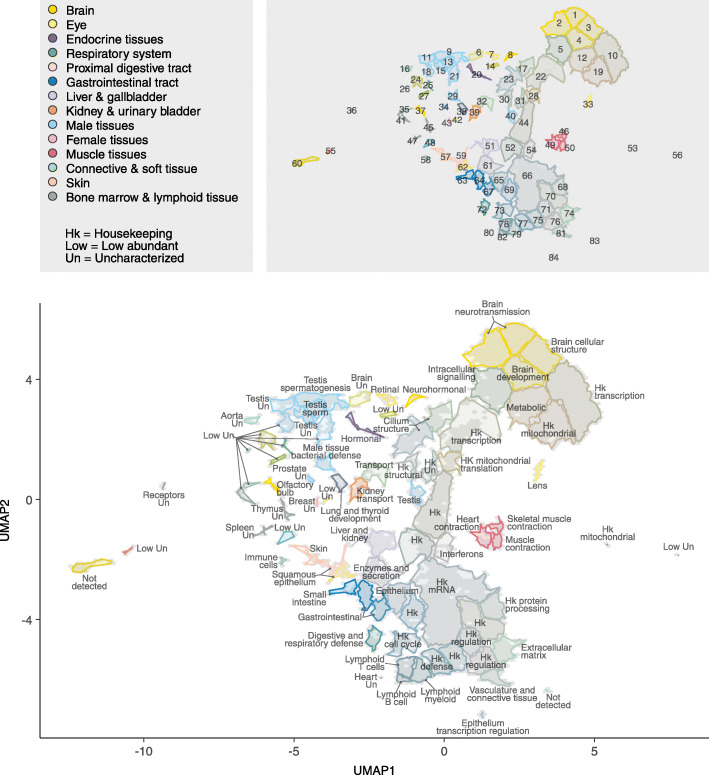
UMAP gene cluster annotation and visualization based on gene expression clustering. UMAP plot showing clustering of 22,342 genes based on their expression in 350 pig tissue samples. The resulting 84 gene clusters are outlined and are color coded by mixing the colors associated with each organ system in proportion to the mean squared fraction of total expression among tissues for genes in the cluster. Top: Color legend and cluster map showing cluster ID numbers. Bottom: Annotated cluster names. See Additional file 1: Fig. S4, for basic gene UMAP visualizations

To complement the genome-wide annotation of expression based on specificity and distribution as previously described, we here introduce a new classification system for gene expression based on dimensional reduction of global expression patterns using UMAP, and subsequently density-based clustering [35]. The expression of 22,342 protein-coding genes across the 350 individual samples was projected onto two dimensions (Additional file 1: Fig S4A-B), and the genes were subsequently classified into 84 clusters based on their expression across the tissues and organs (Fig. 4 and Additional file 4). In this manner, all protein-coding genes have been classified based on their similarity in expression with other genes across all samples, designating each gene into a single Tissue Expression Cluster. Based on the cluster’s expression profile and functional enrichment analyses, an annotation of the clusters was performed, assigning each cluster a name describing the cluster’s specificity, and/or function (Fig. 4 and Additional file 5). To facilitate annotation and further characterize the 84 clusters, tissue specificity category, expression proportion per tissue type, and abundance level were summarized in Additional file 1: Fig. S5. Genes in each cluster can be explored in the open-access Pig RNA Atlas, together with cluster annotations based on Gene Ontology (GO) terms and tissue specificity.

The expression UMAP shows an expression “landscape” with distinct clusters with genes related to tissues and/or functions, such as the testis or muscle contraction. Many genes involved in neurological functions can be found in the brain-related clusters situated adjacent to each other. Similarly, cluster of genes involved in immunological function such as the clusters annotated as “lymphoid B cells,” “lymphoid T cells,” and “housekeeping defence” are found adjacent to each other. Interestingly, the “housekeeping” genes expressed in all tissues are found in distinct clusters, mostly adjacent to each other in the UMAP, as exemplified by the clusters annotated as “housekeeping protein processing” and “housekeeping regulation” (Fig. 4).

When the tissue specificity classification is superimposed upon the cluster landscape (Additional file 1: Fig. S6A), patterns of the various categories emerge. Additional file 1: Fig. S6A shows that genes classified as tissue enriched or group enriched reside in smaller clusters of genes, or at the periphery of larger groups of genes, while genes classified as tissue enhanced are centrally located and partially overlapping with the genes annotated as low tissue specificity. Furthermore, genes classified as tissue elevated in a tissue cluster together, exemplified in Additional file 1: Fig. S6B, which shows how the majority of the genes classified as brain elevated cluster together, spatially distinct from genes classified as elevated in the lung, lymphoid tissues, or testis. In addition to clustering by tissue specificity, genes with a functional relationship can be observed to be co-localized, such as for cluster 23 (a cluster of 477 genes, highlighted in Additional file 1: Fig. S6B), which harbors genes with elevated expression in both testis and lung, as well as choroid plexus, upper respiratory system, and fallopian tube (Additional file 1: Fig. S7), and more in-depth analysis reveals that many of these genes code for proteins of ciliated cells, including proteins involved in mobility, such as the sperm flagella [36].

**Fig. 5:**
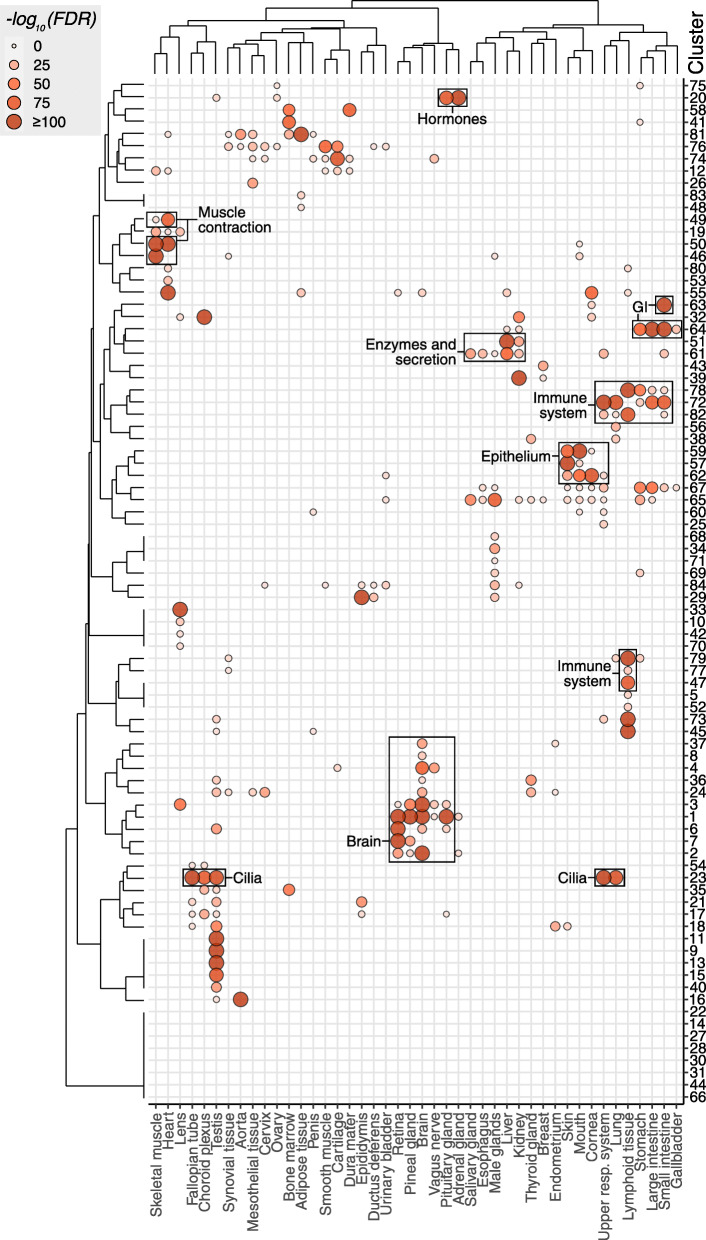
Comparison between UMAP clusters and tissue specificity classification. Bubble heatmap showing the –log_10_(FDR) of the hypergeometric test comparing the overlap of cluster genes with genes classified as elevated in different tissues. FDR values are capped at 10^−100^ to allow for higher contrast in the figure. Only statistically significant overlaps (FDR < 0.001) are shown

To facilitate cluster annotation and find an association between clusters and tissues, a hypergeometric test was conducted, calculating the extent of the observed overlap between genes elevated for each tissue and the cluster genes. Genes classified as elevated in the lung, testis, choroid plexus, upper respiratory system, and fallopian tube are significantly overlapped with cluster 23 (Fig. 5). Indeed, Gene Set Analysis (GSA) towards GO annotations revealed that cluster 23 is enriched with genes related to cilium functions, including cilium movement, organization, and assembly. These results indicate that genes are arranged in groups of clusters with distinct relation to certain tissue types. For instance, clusters 46, 49, and 50 contain genes highly expressed in muscle tissues, although each cluster also shows a distinct expression pattern: cluster 46 is dominated by the skeletal muscle, while cluster 49 is mainly expressed in the heart muscle. Other examples include cluster 33 with almost exclusive expression in the lens and clusters 57 and 59, which include genes important for squamous epithelium and include several different keratin-coding genes.

There are 18 Tissue Expression Clusters containing altogether 9910 protein-coding genes with “housekeeping” functions, with an overrepresentation of genes classified as low tissue specificity, as exemplified by clusters 22, 53, and 66. Functional analysis shows that cluster 66 (2285 genes) is mainly enriched for genes related to transcription, RNA processing, and DNA repair. Similarly, cluster 22 contains 204 genes related to DNA-template regulation of transcription, while cluster 53 only includes 19 mitochondrial protein-coding genes, verifying the housekeeping-related functions of the clusters.

Thirteen clusters were annotated as “low abundant - uncharacterized” due to limited gene information, low expression levels, and limited functional data. However, among the uncharacterized clusters, olfactory receptors were highly represented with cluster 6 harboring 73 out of 88 genes coding for olfactory receptors and cluster 21 with many olfactory receptors (17 out of 54) found in male reproductive tissues, such as the testis and epididymis, and cluster 56 (10 of 13 genes) with olfactory receptors found in the lung and bronchus. This suggests that the porcine olfactory receptors have additional functionality beyond olfaction, which is consistent with previous findings of the human olfactory receptors [37].

In summary, we have introduced a new genome-wide classification scheme to identify genes with similar expression profiles based on dimensional reduction. This has allowed us to classify all pig protein-coding genes into 84 Tissue Expression Clusters. This new approach for classification is an attractive tool for annotation of mammalian proteomes to catalogue all proteins according to body-wide expression patterns.

### Comparison of body-wide gene expression between pig and human

**Fig. 6:**
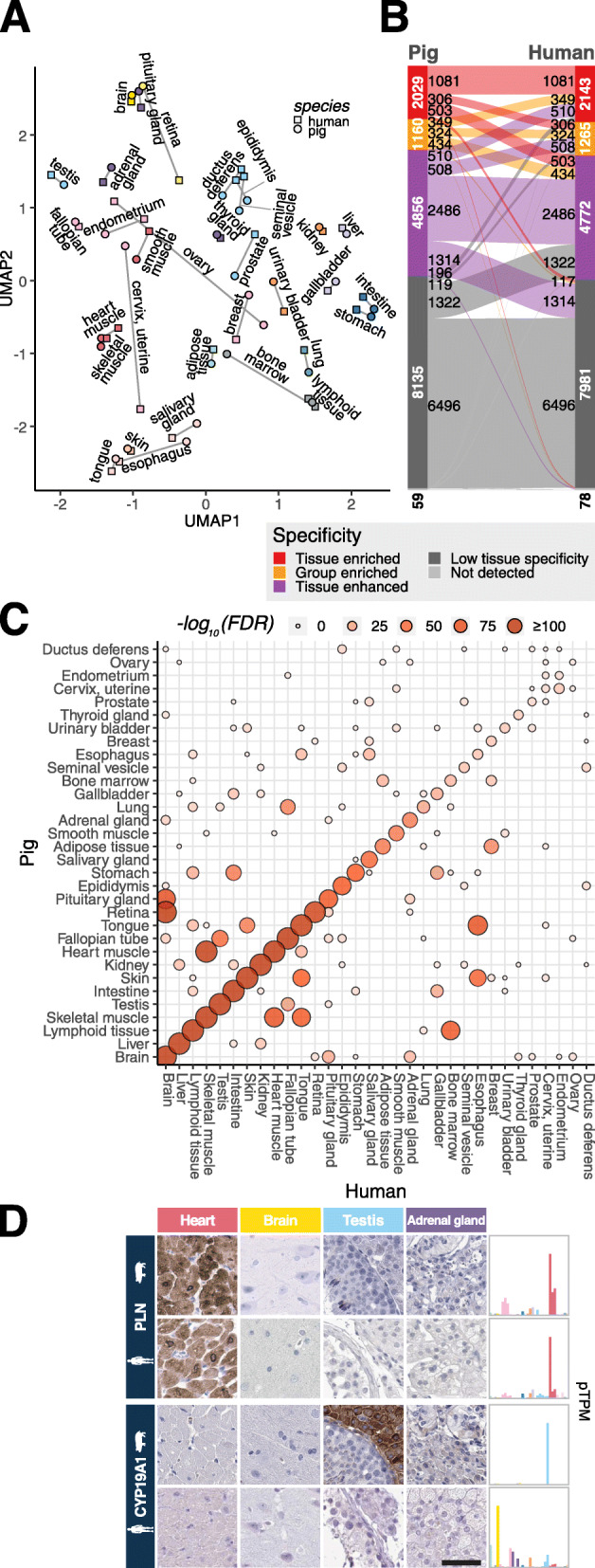
Comparison of gene expression between human and pig. **A** UMAP of human and pig tissues based on expression normalized for ubiquitous expression level differences between species. Lines connect the same tissue between the two species. **B** Alluvial diagram showing the overlap between human and pig, in terms of tissue specificity categories, based on 32 tissue types available in human and pig datasets. **C** Statistical assessment of overlap using a hypergeometric test. The heatmap shows the adjusted *p*-values for statistically significant overlap (FDR < 0.001) between genes classified as tissue elevated in either pig or human tissues. FDR values are capped at 10^−100^ to increase the contrast in the figure. **D** IHC (left) and RNA expression (right) examples: PLN (overlapping expression) and CYP19A1 (not overlapping). The scale bar represents 50 μm

The pig whole-body expression atlas enables us to compare tissue-wide similarities and differences between the pig and human expression. Here, we analyze the expression profiles of 32 tissue types for which the data presented here for pig could be compared with the data already generated for human tissues [1]. First, we generated a UMAP of the global expression profiles of these tissues in human and pig (Fig. 6A). As expected, tissues from the two species cluster together based on tissue types, but certain tissues such as the ovary, breast, and cervix show distinct expression profile differences in pig and human. The retina and bone marrow show a large discrepancy in the clustering, which is expected since the sampling for these tissues from the two species differed. The pig retina was isolated with as little pigment layer as possible, whereas the human retina sample included the pigment layer. Similarly, the pig bone marrow was used without further fractionation, whereas the human bone marrow was Ficoll separated, thus isolating mononuclear cells from e.g. adipose cells, vessels, and non-hematopoietic components [38]. The esophagus and salivary gland also show somewhat different clustering for pig and human tissues, most likely explained by the abundance of glands in the submucosal layer of the pig esophagus, which are limited in the human esophagus.

To achieve a detailed comparison regarding tissue-specific expression profiles, we subsequently investigated the overlap between the specificity classification categories in pig versus human using the updated gene classification described previously [2]. Figure 6B shows that 6496 genes are classified as low tissue specificity in both pig and human tissues, while the remaining 9673 genes are classified as elevated in either of the two species. A majority of the elevated genes are classified similarly in the two species (Additional file 1: Fig. S8B) with few elevated genes showing a different tissue specificity. The gene category overlap was particularly high when comparing tissue enriched and group enriched genes (Additional file 1: Fig. S8B and S8C) with 76% and 80% of the genes having overlap in classification respectively. This demonstrates the similar molecular architecture of these evolutionary close species.

However, there are some interesting differences that are worth more in-depth studies to understand their respective molecular function in human and pig. For example, the neuropeptide galanin (GAL) was classified as tissue enriched in the pig adrenal gland, but was classified as not detected in human adrenal gland samples. Similarly, the pro-neuropeptide Y precursor (NPY) is classified as group enriched in the human adrenal gland, brain, and prostate, while being group enriched in brain and lymphoid tissues in pig. Additionally, the human testis-specific protein, MORC family CW-type zinc finger 1 (MORC1), is classified as enriched in the pig liver. This list of genes classified as elevated in different tissue types between human and pig (Additional file 6) is obviously of high relevance for our understanding of evolutionary processes that drive species differences.

To statistically assess the similarity between human and pig gene classification, a hypergeometric test was performed for each pair of human and pig tissues (Fig. 6C). Brain, liver, and lymphoid tissues show high similarity between human and pig. As expected, the analysis revealed similarities between the heart (cardiac) muscle and skeletal muscle, as well as between the brain and retina. Interestingly, the hypergeometric test suggests overlap in expression profiles between the fallopian tube and lung, which is most likely explained by the presence of ciliated cells in both tissues. To further explore the global transcriptome similarity between human and pig tissues, we performed a genome-wide comparison of gene expression between pig and human for each tissue using Spearman correlation, resulting in 32 scatter plots (Additional file 1: Fig. S8E). The global transcriptome correlation between species for the individual tissue types ranges from 0.60 to 0.80. Collectively, the body-wide gene expression comparison between pig and human thus suggests that the global protein-coding gene expression is similar between the two species. However, an interesting exception is the low similarity for reproductive tissue, as exemplified by ductus deferens, ovary, endometrium, cervix, and prostate. It would be of interest to extend this comparison to other mammals, such as rodents, to give context to the similarity between human and pig.

An alternative approach to investigate similarities and differences between human and pig is to perform antibody-based tissue profiling, to allow a single-cell analysis of the corresponding protein in situ in the context of neighboring cells. Here, we used antibodies raised against the human ortholog to probe the tissue profile in both human and pig tissue (Fig. 6D and Additional file 1: Fig. S8A). The first example is the Phospholamban (PLN) protein showing a similar staining in the heart muscle of both pig and human, supporting its role in calcium regulation in myocytes [39]. Similarly, Cadherin 17 (CDH17) is shown to stain GI-related tissues in both species, supporting the GI-enriched classification in both species. Furthermore, the special AT-rich sequence-binding protein 2 (SATB2) classified as enriched in the intestine and brain in both species shows similar staining in the intestine of both species. It is also reassuring that pyruvate dehydrogenase E1 beta subunit (PDHB) classified as low tissue specificity in both species shows a ubiquitous staining across many tissues in both species (Additional file 1: Fig. S8A).

The antibody-based profiling can also be used to validate the genes with differential expression in the two species. In Fig. 6D, the antibody-based tissue profiling of estrogen synthetase (CYP19A1) is shown. CYP19A1 was classified as testis enriched in pig, but instead enriched in the placenta in humans. The tissue profiling confirms the high abundance of this protein in pig testis, while antibodies to this protein instead stain human placenta [40]. Interestingly, the CYP19A1 catalyzes the synthesis of estrogens from androgens in the steroid hormone biosynthesis and is associated to fertility in pig [41]. In this context, it is interesting to note that many genes related to steroid hormones are differentially expressed in the testis of the two species, most likely due to the abundant number of Leydig cells in pig testis compared to human testis. This is further exemplified by scavenger receptor class B member 1 (SCARB1), a receptor important for uptake of cholesteryl esters and ovarian steroidogenesis [42, 43]. This protein shows a similar protein profile in the adrenal gland, testis, and ovary. However, both the RNA expression level and the protein abundance are much lower in the human testis and ovary as compared to the corresponding tissues in pig (Additional file 1: Fig. S8D).

### The Pig RNA Atlas

An interactive Pig RNA Atlas (www.rnaatlas.org) has been launched as part of this study. This open-access resource harbors more than 20,000 separate web pages, including summary pages for all protein-coding genes of pig. Genes are searchable based on gene name and gene id. Categorizations in terms of specificity, distribution, and UMAP-based Tissue Expression Profile clusters are presented and searchable for each gene. Human ortholog data is an integrative part of the atlas with tissue expression profiles for both human and pig shown on the pig gene summary pages. In addition, the tissues are grouped into organ systems, each described in separate chapters with illustrative images and IHC examples. The Pig RNA Atlas also includes a pig histology dictionary based on representative stained sections from the tissues in this study, providing morphological details and comparison to human tissues.

## Discussion

The pig-centric mammalian transcriptomics map presented here is based on protein-coding expression of 350 samples across 98 well-defined pig tissues divided into 44 tissue groups. The distribution and tissue specificity of gene expression are described for all 22,342 protein-coding pig genes present in Ensembl 92 assembly. Out of these, 18,730 are overlapping with the newer assembly of Ensembl 103, where 413 are reclassified as other gene types than protein coding, mainly pseudogenes (Additional file 8). Interestingly, 232 of the 335 genes classified by us as not detected are removed from the Ensembl 103 assembly. In future versions of the Pig RNA Atlas portal, the data will be continuously updated to later versions in parallel with the update of the human data in the Human Protein Atlas [40]. The classification in both pig and human has allowed a comprehensive comparison of 16,228 gene orthologs in 32 common tissues between pig and human, to decipher the molecular signatures of pig tissues and organs in relationship with the human counterpart, to identify similarities and differences between human and pig.

An important quest for genome biology research is to generate gene-specific annotation based on expression, functionality, and species differences. Efforts such as the UniProt [44], GeneCards [45], GenBank [46], and Ensembl [47] have been important to provide manual or semi-automated annotation of genes. In addition, a large number of expression maps have been described, including the Human Protein Atlas [1, 2, 21], the Human Cell Atlas [48], Gene Expression Atlas [49], and Genotype-Tissue Expression [50, 51] presenting the transcriptome profiles across cells, tissues, and organs of various species and thus contributing to the understanding of biology in humans and other species. The pig transcriptome landscape has previously been described in the context of biomedical research [15] with a large emphasis on muscle and fat tissues due to their importance to industry. Our study has expanded this comparison to a wide variety of tissues with high granularity, including 30 brain regions, endocrine glands, multiple parts of the male and female reproductive system, and lymphoid tissues. In this manner, it has been possible to score individual genes with regard to similar expression patterns across all major tissues and organs in the body. An attempt in this direction was first described as part of the Tissue Atlas [1] in which the *Tissue Specificity*, scored as expression in one tissue compared to all other tissues in the body, was defined for all human protein-coding genes. Later, the Human Protein Atlas annotation was extended to also annotate the *Tissue Distribution* of all genes, scored for a given gene how many tissues the gene can be detected [2]. The fact that these genome-wide annotation tools require arbitrary cut-offs makes it attractive to develop new approaches for genome-wide annotation of expression profiles, without the need to determine fold-change cut-offs or define limits for scoring a gene as “detected.” Here, we describe the use of dimensionality reduction to stratify genes based on similarity of expression patterns across all analyzed tissues, independent of cut-offs. This new strategy allows all protein-coding genes to be annotated as part of a *Tissue Expression Cluster* with relationship to tissue specificity and underlying protein function. This classification has been performed for all 22,342 protein-coding genes of pig and all genes have been classified as part of one out of 84 Tissue Expression Clusters presented for all protein-coding genes in the open-access Pig RNA Atlas, launched as part of this study. A similar gene clustering has previously been performed in a meta-analysis of pig samples from multiple sources [19], where the clustering was performed at a higher granularity, finding 1043 clusters in total, out of which 59 are annotated. Although differences in methodology, samples, and gene inclusion criteria, our clustering produces similar patterns to Summers et al.; 74 of our 84 clusters had a statistically significant overlap to an annotated Summers et al. cluster in a one-sided hypergeometric test considering common gene IDs, with similarities in annotations between overlapping clusters. Clusters annotated by us as “Testis sperm,” “Testis spermatogenesis,” and “Testis uncharacterized” had an overlap to the Summers et al. cluster “Testis only” of 89%, 85%, and 82%, respectively, and “Brain neurotransmission” had an overlap of 72% to “CNS” (Additional file 5). This indicates that independent cluster analyses are able to recreate the main features of expression landscapes.

Although most tissues/organs consist of different cell types, previous studies have shown that most tissues and organs are composed of major cell types from the same germ layers [27]. Previous efforts studying the mammalian transcriptome in mouse cell lines and tissues found that the global transcriptome of these cell types and tissues were clustered according to germ layer origins, including ectoderm (neurectoderm, neural crest, surface ectoderm), endoderm, mesoderm, blood mesoderm, germ cells, and the embryonic domain [52]. In this study, although we do not focus on classifying the germ layer-specific gene expression and distribution, our results (Fig. 1C and Additional file 1: Fig. S1B and S3B) also suggest that tissues derived from the same germ layer are clustered closely.

The comparisons between the pig and human transcriptomes show that a majority of the elevated genes are classified similarly in the two species with very few genes classified as elevated in different tissues. This demonstrates a similar molecular architecture of these evolutionary close related species. It is interesting that most differences are observed for the male and female reproductive tissues. Indeed, the gross anatomy of human and pig uterus is different, e.g., pigs have a bicornuate uterus, as well as a nonseasonal and polyestrous cycle [53], which could contribute to the variance. Furthermore, the pig tissues were sampled at 1 year of age and had not undergone pregnancy, while human samples are sampled from adults of various age (majority above 40 years of age) and unknown previous pregnancy status. Since a previous pregnancy and menopause status both have a considerable impact on reproductive tissues including the mammary, endometrium, and cervix, this could explain some of the differences observed in female reproductive tissues between the human and pig samples. The gene expression difference in reproductive tissues is important both from an evolutionary perspective and also to increase our understanding in the difference of human and pig reproductive biology. While this pairwise comparison between humans and pigs is useful, further research should also investigate similarities across additional species, thereby putting pairwise similarities between species into context, i.e., how similar e.g. pig tissues are to human, in contrast to their similarity to rodent or other primate tissues. This type of effort has previously been published using the brain samples from this study in Sjöstedt et al. [21], which entails a detailed comparison of the pig brain samples to human and mouse brain, highlighting a general species conserved gene expression across brain regions and placing pig closer to the human brain than mouse, based on expression profiles. Furthermore, it would be of additional use to expand the comparison of multiple species to also involve various stages of development, to assess the effect on tissue gene expression.

## Conclusion

In conclusion, we present a new approach for genome-wide functional annotation of protein-coding genes based on UMAP clustering to allow annotation of all pig genes based on an expression analysis, here covering 98 tissues and organs. Comparison of protein-coding transcriptomics supported the evolutionally similarity between pig and human, with some tissues showing higher differences, in particular the reproductive tissues. A genome-wide resource of the transcriptome map across all major tissues and organs in pig has been launched and the data is available as an open-access resource called the Pig RNA Atlas (www.rnaatlas.org) with the expression profile of all protein-coding genes across all tissues, including a comparison to the human orthologs. This resource will facilitate future attempts to understand pig biology and to use pig as a model system for human health and disease.

## Methods

### Pig and sample collection

Sample collection and handling of animals were carried out in accordance with national guidance for large experimental animals and under the permission of the local ethical committee. Four Chinese Bama minipigs (2 male and 2 females, 1 year old) were provided by the Pearl Lab Animal Sci & Tech Co., Ltd. Animals were housed in a specific-pathogen-free pigsty under standard condition. The pigs were deeply anesthetized and sacrificed by terminal bleeding. The sampling order was similar between the four pigs: the peritoneum, abdominal fat, and pancreas were sampled first (in spite of this effort, the pancreas did still fail the quality control and is therefore missing from this study), followed by removal of the eye and orbital adipose tissue. The eye was consequently dissected into the cornea, lens, and retina (with as little pigment layer as possible). After removal of the eye, the skull was opened and the brain was removed. For brain sample processing, the dura mater was sampled and the entire pig brain was removed from the skull and submerged into ice-cold PBS buffer for 2 min to stiffen the tissue and remove excess blood. Brain samples were retrieved from the respective hemispheres, in total 30 samples, of which one side was fixed in whole slabs and the other hemisphere was used for regional sampling and RNA extraction. The brain is further described and analyzed in more detail in Sjöstedt et al. [21]. In summary, the cerebellum and cerebral cortex were collected as pieces while the remaining brain regions and subregions were collected in their entirety.

Peripheral tissue samples were divided into two pieces, one for fixative and morphological verification while the other piece was snap frozen for RNA sequencing. Tissue samples for RNA sequencing were snap frozen in dry ice and stored at − 80 °C until further processing. For tissue fixation, samples were immersion-fixed in phosphate-buffered saline containing 4% paraformaldehyde for 1 week followed by storage of 70% ethanol at 4 °C. The joint cartilage, synovial tissue, and larynx were exceptions without fixed tissue samples, due to being sparse and difficult tissues to sample. The brain samples were stored in phosphate-buffered saline containing 0.1% sodium azide at 4 °C. All samples included in the Pig RNA Atlas are listed in Additional file 2.

### Library preparation and sequencing

RNA extraction was performed with a Trizol-based tissue RNA extraction protocol. The tissue was homogenized mechanically using a pre-cooled Dounce tissue grinder in liquid nitrogen. Total RNA was then extracted with a standardized protocol based on TRIzol reagent (Invitrogen). Quality and quantity of total RNA samples were measured with Agilent 2100 BioAnalyzer (Agilent Technologies). Library preparation was carried out using the DNBseq technology provided by MGI Tech Ltd. First, total mRNA and noncoding RNAs were enriched by removing ribosomal RNA (rRNA) using a MGIEasy rRNA depletion kit (MGI Tech, China). Enriched RNAs were then mixed with RNA fragmentation buffer resulting in short fragments (180 to 300 base pairs). Third, complementary DNA (cDNA) was synthesized from the fragmented RNAs using N6 random primers, followed by end repair and ligation to BGIseq sequencer compatible adapters. The quality and quantity of the cDNA libraries were assessed using Agilent 2100 BioAnalyzer (Agilent Technologies). Finally, the libraries were sequenced on the BGISEQ-500 with 100-bp paired-end read (PE100). A few randomly selected libraries were also re-sequenced and co-validated with the MGI2000 sequencer. An average of 165.5 million reads per samples were generated for each library. Sequencing reads that contained adapters, had low quality, or aligned to rRNA were filtered before following bioinformatics analysis.

### Raw data processing

The output analysis was performed using Kallisto v.0.43.1 [54] and mapped to the pig Ensembl build 92 with 22,342 protein-coding genes, for the initial analysis. An overview of the total reads, Q30 clean reads, and mapping ratio to the pig genome (Sscrofa11.1) is provided in Additional file 2.

### Data normalization

The transcriptomics data were normalized as in a previous publication [2]. In brief, transcripts per million (TPM) values were calculated per each sample (*n* = 350) for all protein-coding genes, referred to as pTPM. Samples of the same tissue type (*n* = 98) were then aggregated by using the average pTPM per gene, and resulting values were sample-wise corrected using trimmed mean of M values (TMM) [55] and then gene-wise pareto scaled (dividing by the square root of the standard deviation), resulting in an expression score referred to as NX. Expression values for grouped tissues were calculated as the maximum expression of sub-tissues. Both TMM-corrected and NX values were used in down-stream analyses, as specified in each section below.

### Gene distribution and specificity classification

Each gene was individually classified in terms of specificity and distribution based on relative NX expression values between 44 different tissue types. The specificity categories were defined as follows: tissue enriched: a single tissue has 4-fold or higher NX than any other tissue, group enriched: 2–5 tissues have NX larger than a fourth of the maximum NX and their average NX is 4-fold higher than any other tissue, tissue enhanced: the gene is neither tissue enriched nor group enriched and one or multiple tissues have an NX at least 4-fold higher than the average NX, low tissue specificity: the gene is neither tissue enriched, group enriched, nor tissue enhanced and detected above cut-off in at least one tissue. The pig gene expression distribution categories were defined as follows: detected in all: NX ≥ 1 for all tissues, detected in many: NX ≥ 1 for at least 31% (*n* = 14) tissues but not in all, detected in some: NX ≥ 1 for more than 1 tissue but less than 31% (*n* = 14), and detected in single: NX ≥ 1 for a single tissue. A gene was classified as not detected if no tissue had NX ≥ 1.

### Network of tissue enriched and group enriched genes

For all genes classified as either tissue enriched or group enriched, the number of genes was calculated for each unique combination of elevated tissues observed, thus forming tissue nodes, as well as gene nodes, the latter indicating the number of elevated genes with elevated expression in the connected tissues. This was visualized in Cytoscape (v 3.6.1) [56], and gene nodes were filtered to remove complexity such that gene nodes were displayed if they (1) contained tissue-enriched genes, (2) contained at least 5 genes, or (3) ranked top 2 largest node for any connected tissue and contain at least 2 genes. The layout was manually curated such that no nodes or edges overlapped.

### Hierarchical clustering

Hierarchical clustering was used in several figures to facilitate data visualization.

Figure 1C: Spearman correlation was calculated between all tissue types, after which the correlation was transformed into a distance (1 – Spearman’s *ρ*). The correlation distance values were then clustered using Ward’s criterion, and the resulting dendrogram was then transformed into a hierarchical graph, where link distances were normalized to emphasize graph connections rather than link distances. Link width is proportional to the distance from the root and is colored according to organ system if only one organ system is present among connected tissue type leaves.

Figure S1B: Spearman correlation was calculated between all tissue types, after which the correlation was transformed into a distance (1 – Spearman’s *ρ*). The correlation distance values were then clustered using the average distance (unweighted pair group method with arithmetic mean).

Figure 5 and S5: Tissues and clusters were both clustered using binary distance of –log10(*p*-value) and Ward’s criterion.

### Comparison of pig and human orthologs

Human orthologs were used for cross-species comparison of gene specificity classification and tissue-wide expression based on transcriptomics data. The analyses were based on pig genes having a human ortholog in Ensembl release 92, and the orthologs included were the one2one orthologs (*n* = 15,483) and the set of one2many orthologs having a single high-confidence pair (*n* = 756). Many2many orthologs and one2many orthologs with only low-confidence pairs or several high-confidence pairs were excluded since the analyses rely on gene-to-gene comparisons. A complete list of ortholog mappings can be found in Additional file 6.

### Gene classification by UMAP analysis

Genes were clustered based on their expression in all samples in order to stratify them into groups with related expression pattern and function, such that global transcriptomic structures can easily be navigated. In doing so, manual decisions in clustering were made such that the number of clusters was reasonably low (*n* = 84), and their average size was neither too large nor too small. The resulting clustering favors accessibility and visualization, rather than optimizing for a particular metric.

Sample-wise TMM-corrected data was log-transformed (log10(TMM + 1)) and transformed to *Z*-score. *Z*-scores of genes with a standard deviation of zero were set to zero. The *Z*-scores were used to create a two-dimensional Uniform Manifold Approximation and Projection (UMAP) [35] using the UMAP implementation in the uwot R package (v 0.1.8), with the parameters n_neighbors = 15, scale = “none”, n_epochs = 1000. The two-dimensional UMAP was then used to cluster genes in two steps: (1) density-based clustering and (2) k-means clustering. The density-based clustering was performed using the algorithm Density based clustering using the density reachability and connectivity clustering (DBSCAN) [57] implementation in the fpc R package (v 2.2-8), and the reachability distance parameter eps = 0.1. This clustering was used in order to define initial *k*-means centers that are evenly distributed in spatially distinct clusters of genes. The two largest clusters were given additional k-means centers by random sampling of gene UMAP coordinates in proportion to their approximate area. Using the now acquired k-means centers, the genes were once again clustered, now using k-means in 50 iterations. Resulting clusters were investigated for enrichment of elevated genes in individual clusters using a hypergeometric test (see “hypergeometric tests”), in order to annotate each cluster based on their tissue specificity. Clusters that showed significant enrichment (Benjamini-Hochberg adjusted *p*-value < 0.05) for the same tissues and were also adjacent were merged to reduce the number of redundant clusters, resulting in the final 84 clusters. The final clusters were again investigated for tissue enrichment using the same hypergeometric test, where a cluster-tissue overlap was considered significant for Benjamini-Hochberg-adjusted *p*-values < 0.001.

### UMAP cluster annotation

To functionally annotate the gene clusters from UMAP analysis, gene ontology analysis was carried out using the enrichR R package (v 2.1) [58]. For each cluster, pig genes were transferred to human gene names using the established orthologs (see the section “Comparison of pig and human orthologs” above) and analyzed for enrichment to GO 2018 databases: Biological Process, Cellular Component, and Molecular Function. The result was filtered such that GO terms with adjusted *p*-value (provided by enrichR) below 0.05 were kept. The remaining GO terms were summarized and visualized using rrvgo (v 1.0.1) and treemapify (v 2.5.3) and visualized together with tissue specificity hypergeometric test results and an expression heatmap of cluster genes. This data, together with manual investigation of genes, were used to manually annotate each cluster in terms of specificity and function where possible. The final list of cluster annotations can be found in Additional file 4.

### Human sequencing datasets

Human data was acquired from v 19 of the HPA (http://v19.proteinatlas.org/about/download), which includes expression data from The Functional Annotation of Mammalian Genomes 5 (FANTOM5) project [59] and the Genotype-tissue Expression (GTEx) project [51, 60, 61] normalized according to a previous publication [2].

### Hypergeometric tests

Hypergeometric tests were performed using phyper (stats v 4.0.2) [62] with the following parameters for testing tissue specificity—MAP cluster overlap, and overlap of tissue specificity in human and pig, respectively:

UMAP cluster versus pig tissue specificity: The test was used to evaluate the overlap between genes elevated for a particular tissue and genes within a particular cluster; *q*—the number of genes both elevated for the tissue, and present in the cluster; *m*—the total number of genes in the cluster; *n*—the total number of pig genes; *k*—the number of genes in the cluster. The analysis was repeated for all observed combinations of cluster affiliation and tissue elevation.

Human tissue specificity vs pig tissue specificity: The test was used to evaluate the overlap between established orthologs elevated in a particular human tissue and a particular pig tissue; *q*—the number of orthologs elevated in both the human and pig tissue; *m*—the minimum number of orthologs elevated in human or pig tissue; *n*—the total number of orthologs; *k*—the number of orthologs elevated in both or either the human and pig tissue. The analysis was repeated for all observed combinations of human and pig elevated tissues among orthologs.

### Tissue processing

Peripheral tissues were stored in 70% ethanol at 4 °C while brain tissues that were stored in PBS buffer at 4 °C were moved into ethanol 1 week prior to paraffin embedding. Dehydration in absolute alcohol (VWR chemicals) and xylene (Histolab) followed by paraffin (Histolab) immersion were performed using an automated Tissue Processing Center TPC 15 Duo (MEDITE) machine. After manual embedding into separate paraffin tissue blocks, one representative section (4um) was taken from each tissue block using microtome (Microm HM 355S, Thermo Fisher Scientific) with a microm STS Section-Transfer-System (waterfall) for section transfer into warm water bath (38 °C) stretching before placed on SuperFrost PlusTM slides (VWR). Slides were dried in room temperature for 24 h followed by 50 °C over night (LAMB Windsor Incubator E18.31, Histolab).

### Tissue staining

The representative section for each tissue block was stained with hematoxylin and eosin (H&E) for morphological examination, as well as digitalization for the online tissue dictionary (www.rnaatlas.org/pig/dictionary). Deparaffinization and rehydration of tissue slides were performed using Leica ST5010 Autostainer XL starting with 11 min (5 + 5 + 1) incubation in xylene (Histolab), followed by 6 min (3 + 3) ethanol absolute (VWR), 8 min (5 + 3) 96% ethanol (VWR), 3 min 80% ethanol (mixed from 96% ethanol), and finally 3 min deionized water. The rehydration steps were followed by 3 min in filtered Mayers hematoxylin (Histolab), 3 min wash in running water, 1 min in lithium carbonate (Merck Millipore, 1:5 saturated lithium carbonate in deionized water), another 5 min in running water, and then 1 min Eosin Y before initiation of dehydration and cover glass mounting. Dehydration was performed by 14 s (7 + 7) 80% ethanol, 14 s (7 + 7) 96% ethanol, absolute ethanol (7 s + 3 min + 3 min), and 6 min (3 + 3) NeoClear® before automated cover glass (VWR) mounting by the Autostainer XL extension (slide mounting robot Leica CV5030 unit) with Pertex® (Histolab) as mounting media. Slides were moved from the mounting rack into an oven for drying two times 20 min at 50 °C.

### TMA construction

A selection of tissue blocks, representing most normal tissue types, was used for creating a tissue microarray (TMA) using semi-automated TMAarrayerTM (Pathology Devices). The final TMA included 32 different tissues from Pig 1 (female) and 4 (male), represented by 1-mm punches moved from the donor block to an empty recipient paraffin block according to the previously described method [63]. The TMA block was cut following the identical details described above and stored at −20 °C until used for staining. The TMA slides were used for IHC staining, providing simultaneous results for a large number of tissue types for comparison to RNA expression profile for the corresponding tissues.

### Antibody resource

The resource of antibodies produced in the HPA project was utilized for protein profiling of pig tissues. Genes of interest were investigated in the internal Laboratory Information Management System (LIMS) based on two important criteria: antibodies with high reliability (based on human antibody validation [64, 65]), and over 80% sequence homology between the PrEST (protein epitope signature tag, used for immunization [66]) and corresponding pig orthologous gene. The access to the exact amino acid (aa) sequence for the antigens (PrEST) used for immunization enables the comparison to pig sequence for corresponding orthologs. The exact aa- sequence for each antibody presented in this study is listed in Additional file 7along with the % homology to the pig gene as well as antibody concentration and dilution used for the IHC protocol. All antibodies are published on the HPA portal (www.proteinatlas.org) with more details about antibody reliability and tissue distribution in human. Selected antibodies were first confirmed on human tissues, reproducing the online human staining profile, before applied on the pig tissue, using the exact same pretreatment and staining protocol used for the human tissues within the HPA standardized pipeline.

### Immunohistochemical staining protocol

Deparaffinization and rehydration were performed by Autostainer XL (ST5010, Leica biosystems) as described above by exiting the program after 30 s in deionized water, as well as the addition of 1:100 0.3% H2O2 (Merck Milipore) to the 5 min 96% ethanol (VWR) incubation for blocking endogenous peroxidase. Slides were placed in deionized water before being changed into retrieval buffer. Heat-induced epitope retrieval was done in pH 6.1 citrate buffer (DAKO, diluted 1:10 with deionized water and stored at 4 °C) and pressure boiler (decloaking chamber, Biocare Medical) preheated to 80 °C. The total heating program is 35 min, first heating to 125 °C and stays at 125 °C for 4 min followed by passive cooling to 99 °C and then active cooling (fan) to 90 °C. Slides were then cooled off by running deionized water in the sink for a few min and then placed in wash buffer. The wash buffer is mixed by 9.5 l deionized water, 0.5 l Tris-buffered saline, and tween 20 (ThermoFisher Scientific) and 15 ml large volume tween 20 (ThermoFisher Scientific).

Autostainer 480 (ThermoFisher Scientific) was used for automated IHC staining with UltraVision™ Quanto Detection System HRP DAB-kit from Thermo Fisher Scientific including; Ultra V Block, HRP Polymer, Primary Antibody Enhancer, DAB Quanto Substrate, DAB Quanto Chromogen and primary antibodies were diluted in Antibody Diluent OP Quanto. Rinsing between incubations was done using the wash buffer except for the final step, after DAB Quanto incubation, where deionized water was used for rinsing. All in-house HPA antibodies are affinity-purified polyclonal rabbit antibodies; the antibody production has been described in detail previously [67]. The secondary HRP Polymer (ThermoFisher Scientific) is anti-rabbit, and for this reason, 20 min Primary Antibody Enhancer (ThermoFisher Scientific) was added prior to the HRP polymer incubation in the protocol of anti-SATB2 (AMAb90682), which is a monoclonal mouse antibody. In all other cases, the IHC protocol was identical; first, a rinse with wash buffer followed by 5 min Ultra V Block, rinse twice, primary antibody incubation for 30 min, rinse twice, HRP Polymer incubation for 30 min, rinse twice, 5 min DAB Quanto incubation and then a final rinse with deionized water. Slides were placed in water and moved to the Autostainer XL (ST5010, Leica biosystems) for counterstaining (hematoxylin), dehydration, and cover glass mounting. Slides were incubated 7.5 min in filtered Mayers hematoxylin (Histolab) followed by 5 min wash in running water, 1 min in lithium carbonate (Merck Millipore, 1:5 saturated lithium carbonate in deionized water), another 5 min in running water. Dehydration was performed by 6 min (3 + 3) 80% ethanol, 6 min (3 + 3) 96% ethanol, 9 min (3 + 3 + 3) ethanol absolute, and 6 min (3 + 3) NeoClear® before cover glass (VWR) was automatically mounted by the slide mounting robot Leica CV5030 unit using Pertex® (Histolab) and then dried in the oven for 40 min at 50 °C. Image digitalization was performed with Scanscope AT2 (Aperio) using a 20× objective.

### Data visualization

Data visualization was performed in R (v 4.0.2) [62], using RStudio (v 1.3.1093) [68]. The majority of visualizations were made using ggplot2 (v 3.3.2) [69], supplemented with the following packages: concaveman (v 1.1.0), dendextend (v 1.14.0) [70], ellipse (v 0.4.2), ggalluvial (v 0.12.2) [71], ggalt (v 0.4.0), ggbeeswarm (v 0.6.0), ggdendro (v 0.1.22), ggforce (v 0.3.2), ggraph (v 2.0.3), ggrepel (v 0.8.2), ggridges (v 0.5.2), ggthemes (v 4.2.0), igraph (v 1.2.6) [72], patchwork (v 1.0.1), pcaMethods (v 1.80.0) [73], pheatmap (v 1.0.12), rrvgo (v 1.0.1), sf (v 0.9-6) [74], tidygraph (v 1.2.0), treemapify (v 2.5.3), umap (v 0.2.6.0) [35], uwot (v 0.1.8), and viridis (v 0.5.1). Cytoscape (v 3.6.1) [56] was used to adjust network visualizations. Figures were assembled, annotated, and aesthetically adjusted in Affinity designer (v 1.8.5.703).

## Supplementary Information

**Additional file 1.** Supplementary Figures S1 – S8

**Additional file 2.** List of sequencing quality control metrics, overview of samples used for analysis, and a description of the mapping of tissue types to organ systems and grouped tissues.

**Additional file 3.** Stratification of body-wide distribution and specificity of pig genes, including a list of specificity and distribution classification for all porcine genes.

**Additional file 4.** List of genes in each UMAP cluster, as well as their specificity and distribution classification, and result from hypergeometric test for overlap in tissue specificity and cluster genes.

**Additional file 5.** Supplementary information for annotation of each UMAP cluster, including elevated tissues generated from the hypergeometric test (FDR < 0.001 in sheet 3; Cluster tissue hypergeom), and list of gene set enrichment analysis (GSEA) analysis towards GO-terms.

**Additional file 6.** Includes a list of a subset of orthologs with one parsimonious high confidence ortholog used in human-pig comparison analyses, a table of specificity classification for human and pig, indicating overlap in classification, and the inputs for hypergeometric test for assessing the classification overlap between pig and human tissues.

**Additional file 7.** Includes a table indicating which antibodies have been used in each figure, table of antibody information including ID, targeted gene, and antigen sequence, and a table of staining reagents.

**Additional file 8.** Includes a gene-wise comparison of Ensembl 92 and 103 gene IDs with comments on changes in gene type and status.

## Data Availability

The dataset supporting the conclusions of this article is available in the download section of the Pig RNA Atlas (www.rnaatlas.org/about/download), and in addition, normalized and processed expression data are visualized at individual gene summary pages (e.g., www.rnaatlas.org/ENSSSCG00000017343-GFAP). Pig RNA sequencing data generated in this study have been deposited to the public data depository CNGB Nucleotide Sequence Archive (CNSA; https://db.cngb.org/cnsa/) of the China National GeneBank DataBase (CNGBdb) with accession number CNP0001361. Pig Brain RNA sequencing data are available under the accession number: CNP0000483. The human RNA sequencing data is available in the download section of the Human Protein Atlas (www.proteinatlas.org/download). H&E-stained images are available in the tissue dictionary from the Pig RNA Atlas resources (www.rnaatlas.org/dictionary). R scripts used for analysis and visualization are publicly available at www.github.com/maxkarlsson/Pig-Atlas.

## Abbreviations

cDNA: Complementary deoxyribonucleic acid
DNA: Deoxyribonucleic acid
GI: Gastrointestinal
GO: Gene Ontology
GSA: Gene set analysis
H&E: Hematoxylin and eosin
HPA: Human Protein Atlas
IHC: Immunohistochemistry
NX: Normalized expression
PBS: Phosphate-buffered saline
PCA: Principal component analysis
RNA: Ribonucleic acid
rRNA: Ribosomal ribonucleic acid
TMA: Tissue micro array
TMM: Trimmed mean of M values
TPM: Transcripts per million
UMAP: Uniform Manifold Approximation and Projection
