# Supplementary material for: Genome-wide annotation of protein-coding genes in pig
Source: BMC Biol. 2022 Jan 25;20:25. doi: 10.1186/s12915-022-01229-y (PMC8788080; doi:10.1186/s12915-022-01229-y)
Supplement: Supplementary file 1 — Additional file 1. Supplementary Figures S1 – S8 [file 12915_2022_1229_MOESM1_ESM.docx]

**Additional File 1**

**Genome-wide annotation of the protein-coding genes in pig**

Max Karlsson^1^ #, Evelina Sjöstedt^2,3^ #, Per Oksvold^1^, Åsa Sivertsson^1^, Jinrong Huang^4,5,6^, María Bueno Álvez^1^, Muhammad Arif^1^, Xiangyu Li^1^, Lin Lin^6,7^, Jiaying Yu^4,5^, Tao Ma^8^, Fengping Xu^4,5^, Peng Han^5^, Hui Jiang^8^, Adil Mardinoglu^1^, Cheng Zhang ^1^, Kalle von Feilitzen^1^, Xun Xu^4^, Jian Wang^4^, Huanming Yang^4^, Lars Bolund^4,5,6^, Wen Zhong^1^, Linn Fagerberg^1^, Cecilia Lindskog^3^, Fredrik Pontén^3^, Jan Mulder^2^, Yonglun Luo^4,5,6,7, &^ and Mathias Uhlen^1,2, &,^*

^1^Department of Protein Science, Science for Life Laboratory, KTH-Royal Institute of Technology, Stockholm, Sweden

^2^Department of Neuroscience, Karolinska Institutet, Stockholm, Sweden

^3^Department of Immunology, Genetics and Pathology, Uppsala University, Uppsala, Sweden.

^4^BGI-Shenzhen, Shenzhen, China

^5^Lars Bolund Institute of Regenerative Medicine, Qingdao-Europe Advanced Institute for Life Sciences, BGI-Qingdao, Qingdao, China

^6^Department of Biomedicine, Aarhus University, Aarhus, Denmark

^7^Steno Diabetes Center Aarhus, Aarhus University Hospital, Aarhus, Denmark

^8^MGI, BGI-Shenzhen, Shenzhen, China

# Contribute equally

& Contribute equally

* Corresponding author

**This file includes**

Supplementary Figures S1 – S8

**SUPPLEMENTARY FIGURES**


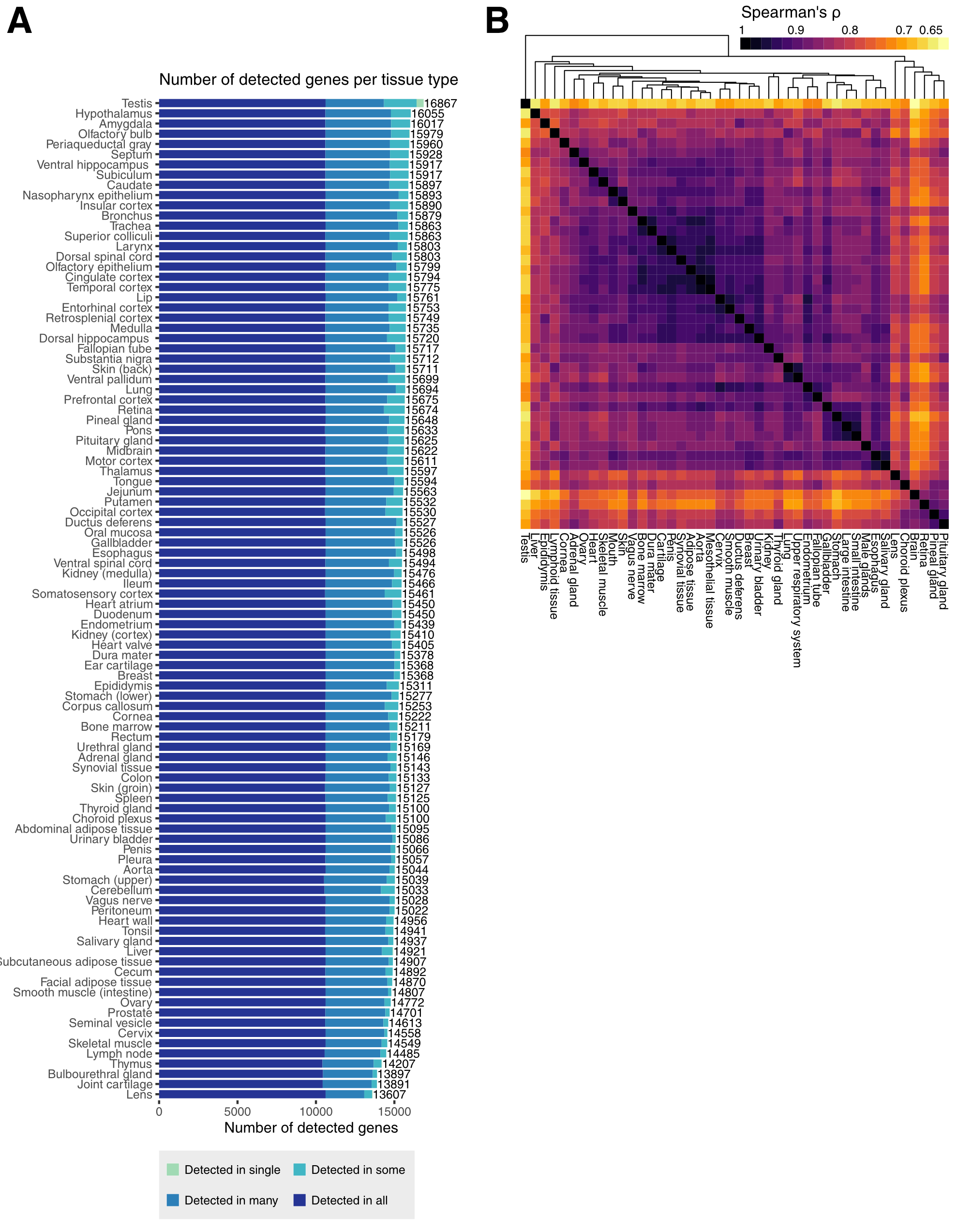


**Figure S1. Global gene expression, Related to Figure 1**

(A) Barplot showing the number of detected genes per each tissue type, sorted based total number of genes above cut-off. Bar segment color indicates the different distribution categories. (B) Heatmap showing the pairwise Spearman correlation of global expression across the 44 different tissue types.


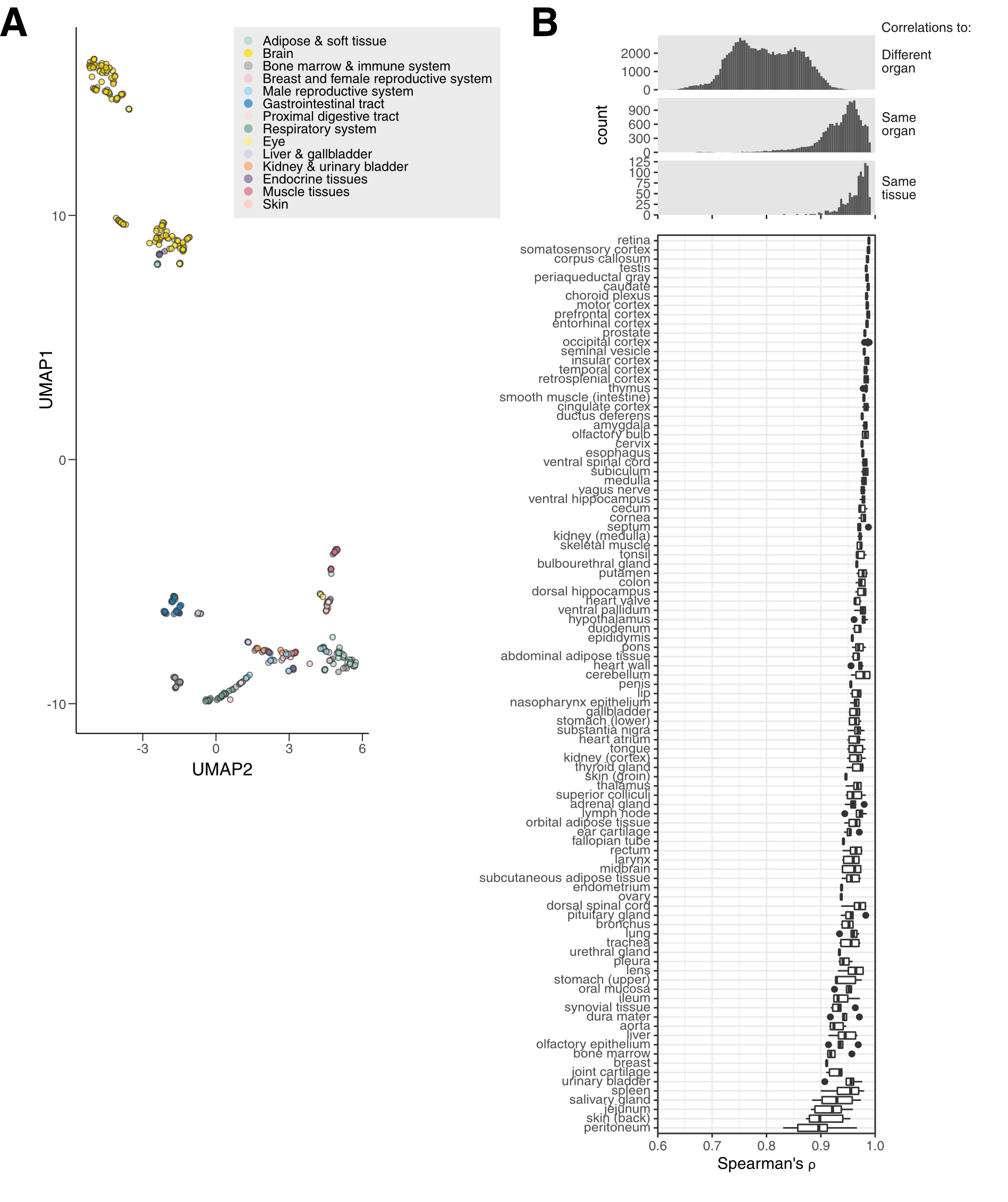


**Figure S2. Sample UMAP and correlation, related to Figure 2**

(A) UMAP visualization illustrating global expression clustering, as an alternative to the PCA in Figure 2. The clustering similarly shows that brain is far from the peripheral tissues, with the exception of vagus nerve, endocrine tissues and eye tissue samples. (B) Top panel: Histograms showing populations of Spearman correlations between samples of different organ systems (top), same organ system (middle), and same tissue type (bottom). Bottom panel: Boxplots showing Spearman correlations between samples of the same tissue type (y-axis).


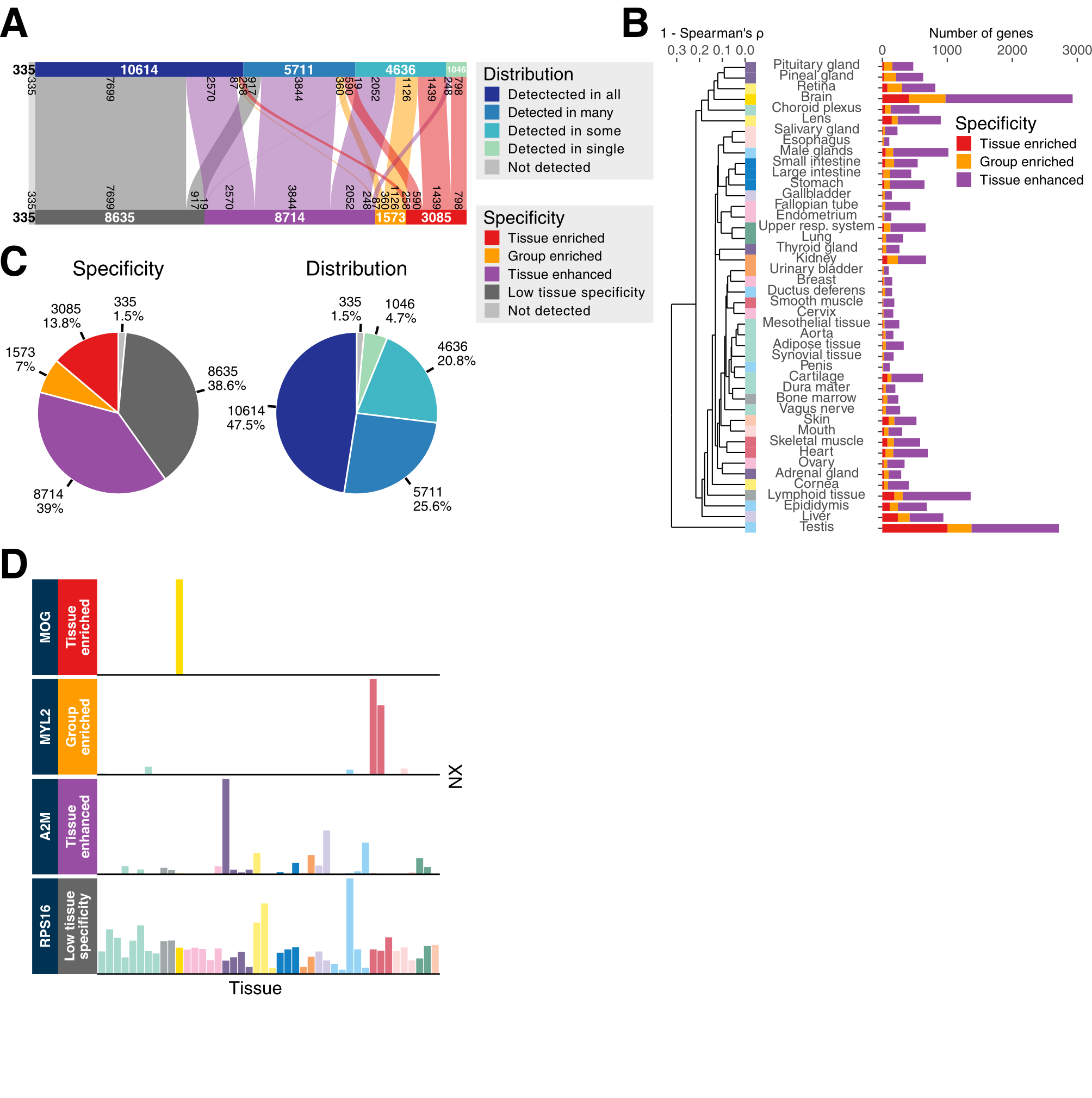


**Figure S3. Gene classification based on tissue expression, related to figure 3**

(A) Alluvial diagram showing the overlap between categories for all protein-coding genes (n=22,342), in terms of tissue specificity and distribution. (B) Dendrogram based on average distance (1 – spearman’s rho) and a barplot of the number of elevated genes for each tissue type. (C) Pie charts showing the number of genes classified per each category of tissue specificity (left), and distribution (right). (D) Examples of expression patterns of the 4 categories of specificity tissue enriched, group enriched, tissue enhanced, and low tissue specificity.


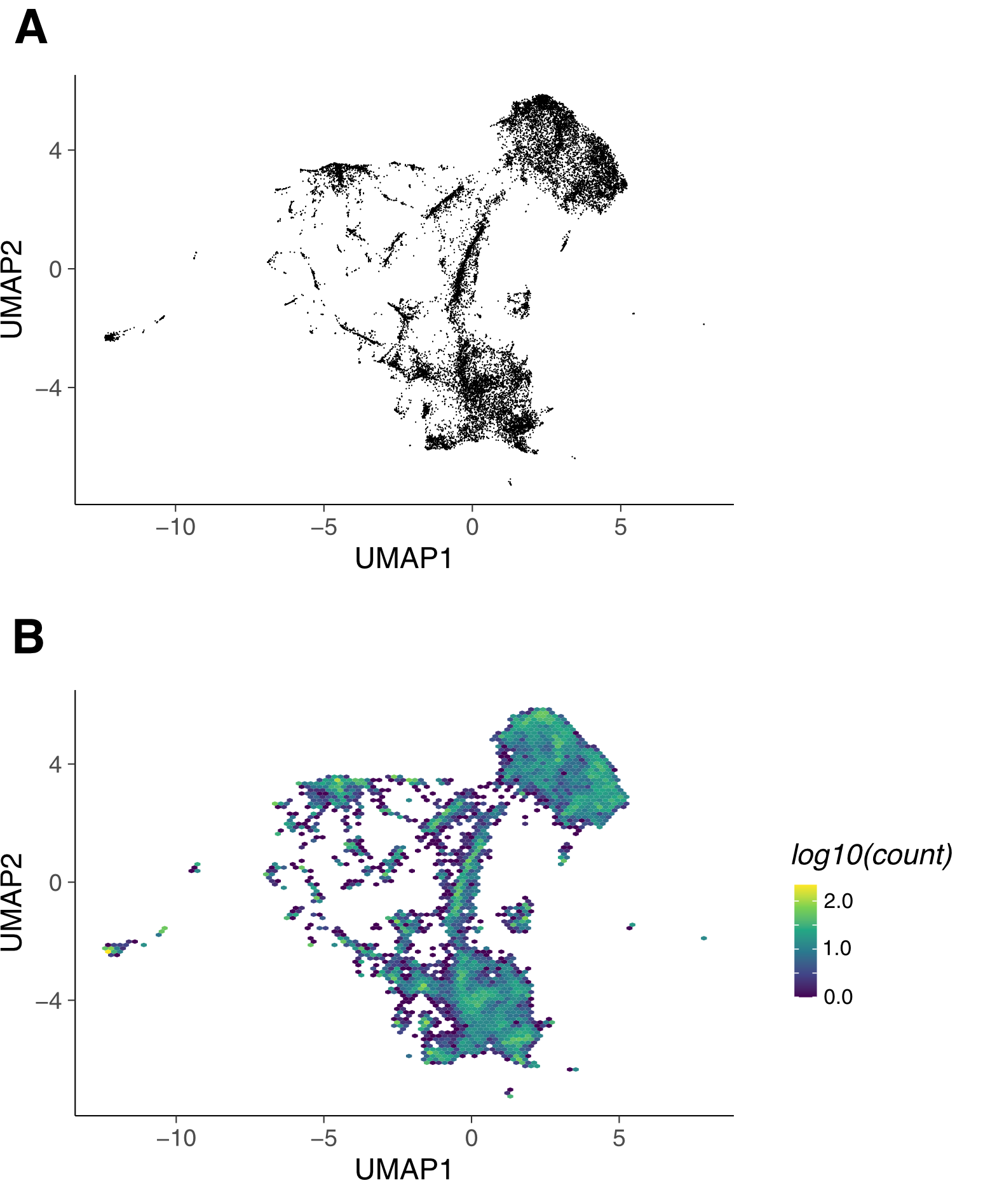


**Figure S4. Gene** **UMAP based on gene expression, related to Figure 4**

Two UMAP variants showing clustering of 22,342 genes. (A) UMAP showing individual points for each gene, and (B) A hexagonal binned density plot showing the log10-scaled gene count underlying each hexagon.


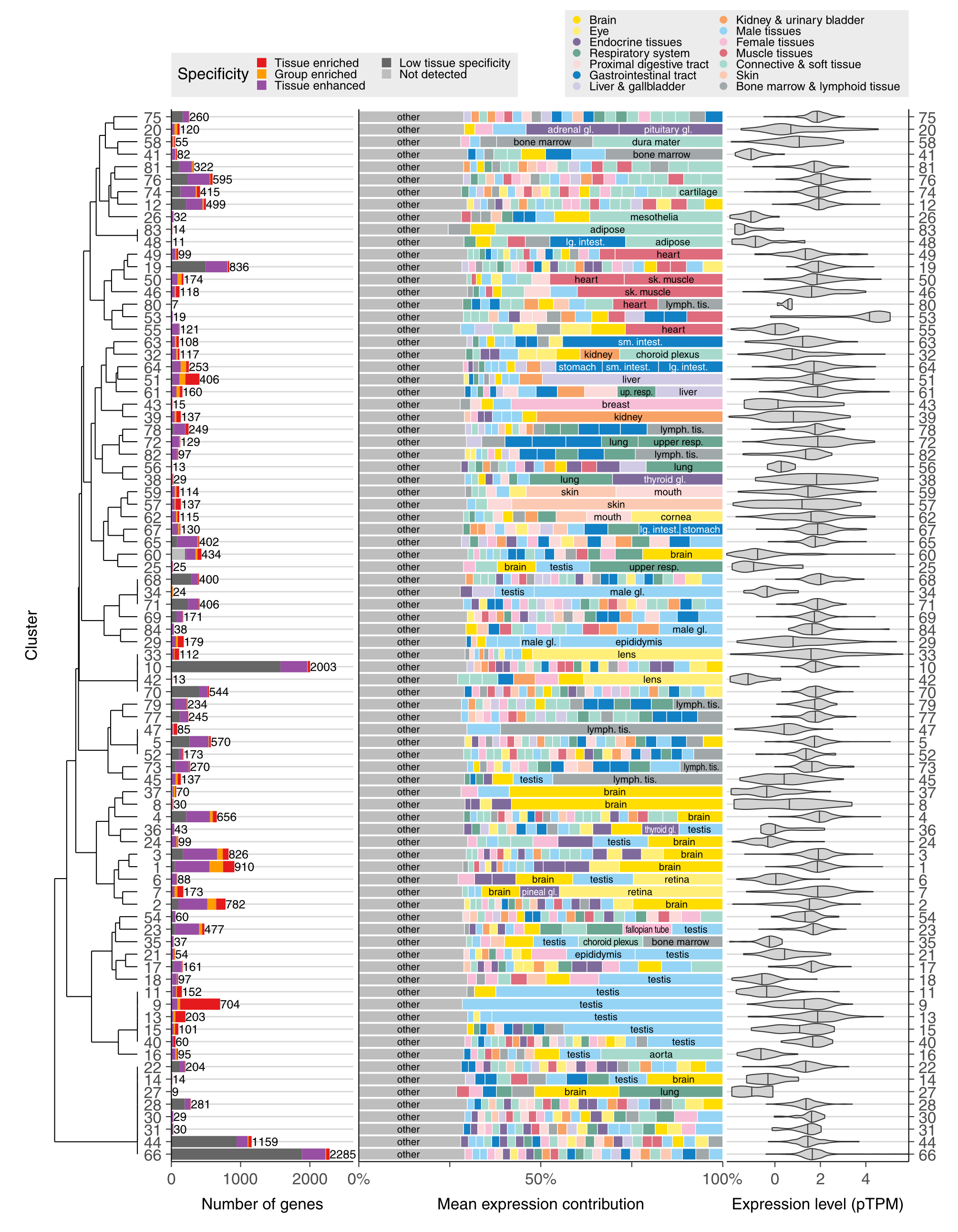


**Figure S5. Summary of UMAP cluster characteristics, related to Figure 4**

Left: Dendrogram from Figure 3D, and a bar plot showing the number of genes for respective cluster and tissue specificity categories. Middle: Stacked barplot showing the mean expression contribution from tissues for the genes in the cluster. Right: Violin plot showing the expression level for the genes of each cluster.


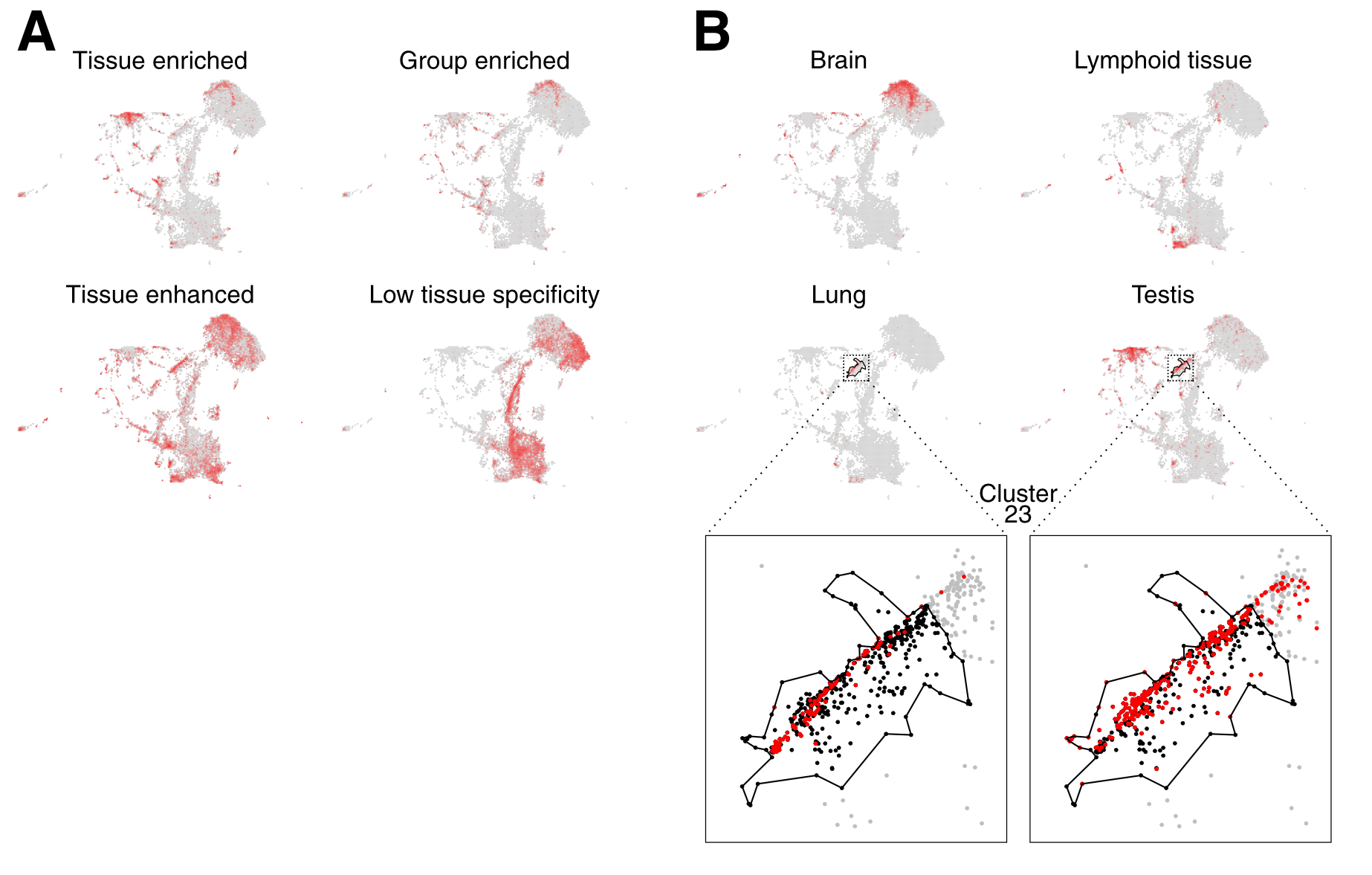


**Figure S6. UMAP visualization of tissue specificity, related to Figure 4 and 5**

Hexagonal binned UMAP density plot, where the intensity of red is proportional to the fraction of genes summarized by the hexagon classified as indicated in the plot title: (A) Projection of tissue specificity categories of genes overlaid on the UMAP. (B) Projection of genes with elevated expression in brain, lymphoid tissue, lung, and testis overlaid on the UMAP. Cluster 23 is highlighted and the bottom of the figure shows an outline of the cluster with genes annotated as specific for lung or testis respectively marked in red.


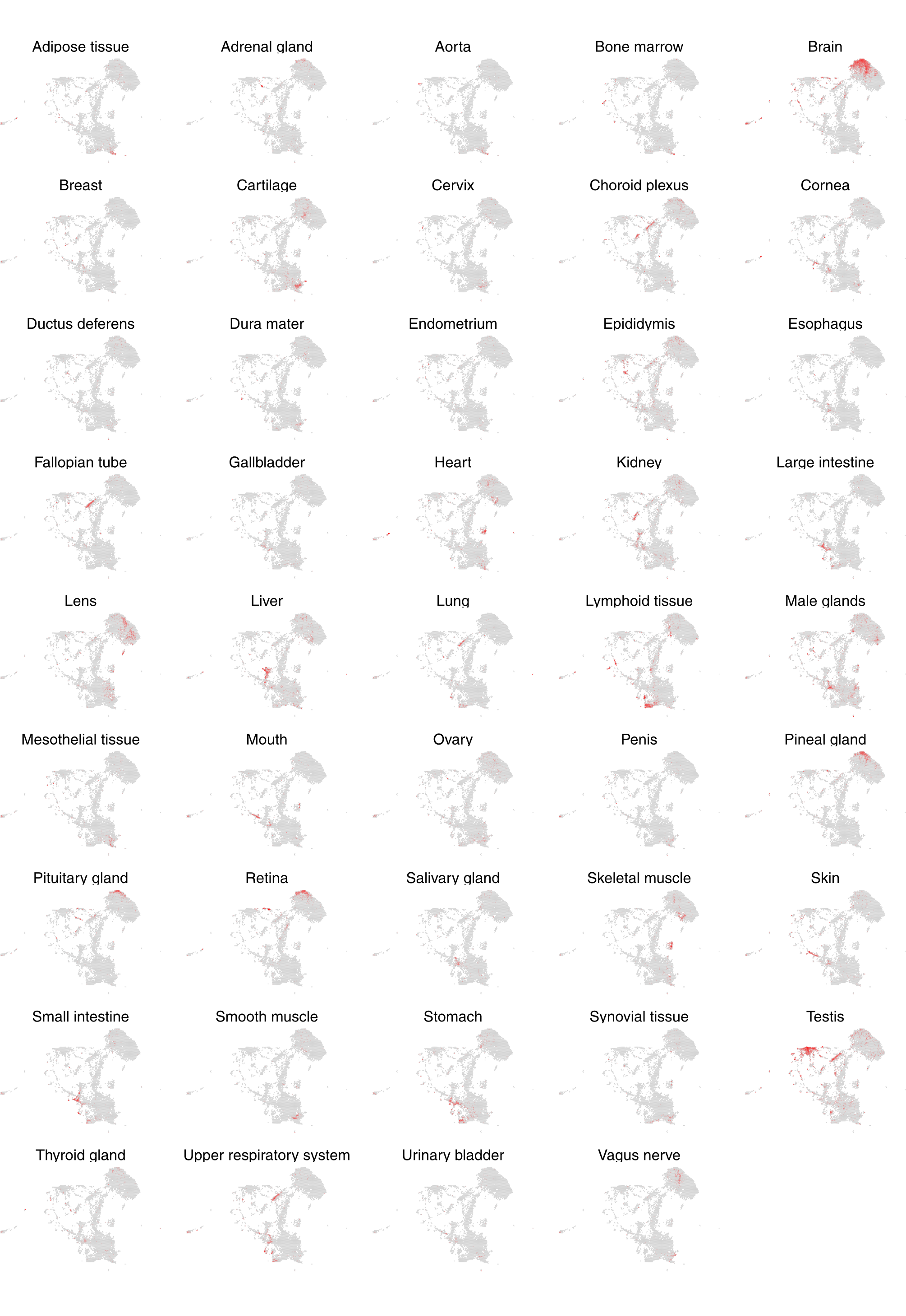


**Figure S7. UMAP visualization of tissue specificity for all tissues, related to Figure 4 and 5**

Hexagonal binned UMAP density plot, where the intensity of red is proportional to the fraction of genes summarized by the hexagon classified as elevated for each tissue.


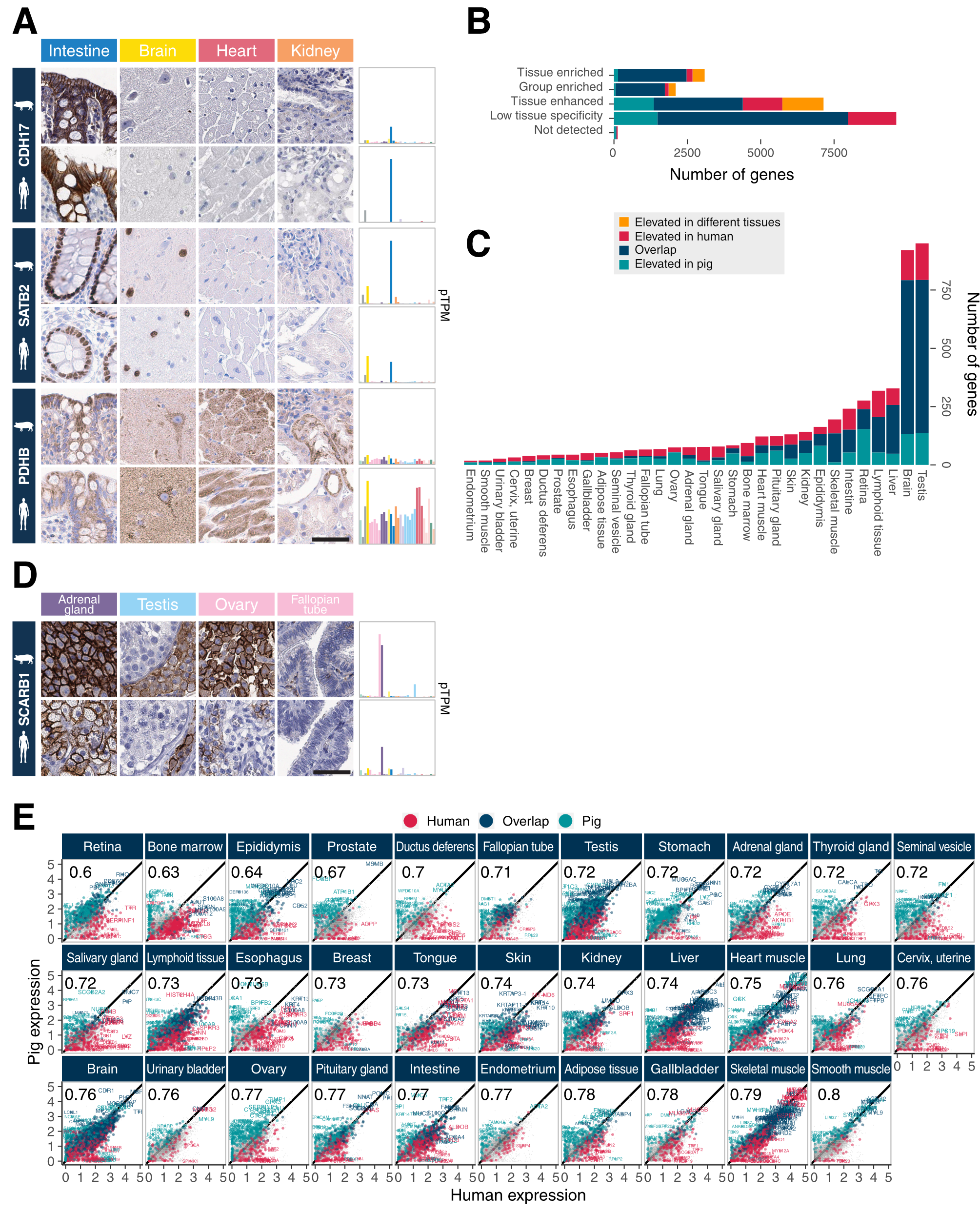


**Figure S8. Comparison of gene expression between human and pig, related to Figure 5**

(A) Immunohistochemical examples of similar protein location in pig and human tissues: CDH17 is specifically detected in the intestine. SATB2 is a transcription factor with group-enriched expression in the brain and large intestine. Mitochondrial protein PDHB is classified as low tissue specificity. The scale bar represents 50 µm. (B) Barplot showing the number of genes with overlap for the different specificity categories. (C) Barplot showing the overlap of genes classified as tissue enriched in either human or pig, per tissue type. (D) Immunohistochemical example of differences in protein expression between pig and human: SCARB1 is classified as adrenal gland enriched in human while the ovary has the highest expression level in pig. The scale bar represents 50 µm. (E) Scatterplot showing the expression correlation for all 16,228 pig genes with human orthologs, in the 32 tissue types represented in pig and human datasets.
